# Test-Retest Reliability and Internal Consistency of the Coronary Artery Disease Education Questionnaire Short Version in the Marathi Language

**DOI:** 10.7759/cureus.32815

**Published:** 2022-12-22

**Authors:** Varoon C Jaiswal, Nalina Gupta, Sumitra Sakhawalkar, Snehalata Tembhurne, Priya Deshpande, Laxmikant Umate

**Affiliations:** 1 Department of Cardiovascular and Respiratory Physiotherapy, College of Physiotherapy, Sumandeep Vidyapeeth, Deemed-to-be-University, Vadodara, IND; 2 Department of Neurophysiotherapy, College of Physiotherapy, Sumandeep Vidyapeeth, Deemed-to-be-University, Vadodara, IND; 3 Department of Neurophysiotherapy, Maharashtra Academy of Engineering and Educational Research’s Physiotherapy College, Talegaon Dabhade, IND; 4 Department of Musculoskeletal Physiotherapy, Maharashtra Academy of Engineering and Educational Research’s Physiotherapy College, Talegaon Dabhade, IND; 5 Department of Statistics, Symbiosis International (Deemed University), Mulshi, IND; 6 Department of Research and Development and Consultant Biostatistician, Jawaharlal Nehru Medical College, Datta Meghe Institute of Medical Sciences, Wardha, IND

**Keywords:** cronbach’s alpha, cohen’s kappa, internal consistency, adaptation, translation, reliability, marathi version, coronary artery disease

## Abstract

Introduction

Patient education specific to the disease must be incorporated into the management of coronary artery disease (CAD) or any other disease as the patients understand the education provided in their native language better. The brief version of the CAD education questionnaire is a valid and reliable instrument for evaluating patients' understanding of the condition. To the best of our knowledge and understanding from the literature review, no questionnaire evaluating the knowledge of CAD in the Marathi language had been found because of which this study was carried out.

Methods

For the process of translation and cross-cultural adaptation, the framework for self-report measures was taken into consideration in which qualified translators translated both ways forward into the Marathi language and backward into the English language. The translators and a recording observer combined their efforts to create one synthesized version. The questionnaire was fine-tuned by the expert group to produce the final version and 30 diagnosed cases of CAD were tested with the pre-final version. The Marathi version of the questionnaire's validity and reliability were evaluated using Cohen's kappa (k) and Cronbach's alpha (α).

Results

Thirty individuals with CAD were recruited (mean age 68±12.36, consisting of 22 males and 08 females) to test the pre-final version, and equivalence was tested for every item by probing the participants for the understanding of the item. The Likert scale demonstrated that patients understood the purpose of each question. A total of 200 participants - 153 males population and 47 females with a mean age of 66.64±5.6094 years who can read and speak in the Marathi language were considered to assess the test-retest reliability and internal consistency who completed the questionnaire twice, with a gap of two weeks but only 188 participant's data was analyzed as twelve participants dropped out of the study because they could not report due to transportation and health-related issues. The obtained α value demonstrated satisfactory internal consistency, while the k value indicated almost perfect agreement.

Conclusion

The study concluded that the Marathi version of the CAD education questionnaire short version is reliable and cross-culturally adapted; therefore, it is an effective tool for evaluating the knowledge of CAD among Marathi language-speaking patients.

## Introduction

Coronary artery disease (CAD) is one of the major causes of mortality and disability [1-3]. As per the American Heart Association, CAD is responsible for one-third of all fatalities worldwide [4]. The most common reason for mortality and impairment in India, between 1990 and 2016, is CAD [5]. The major causes of death worldwide, accounting for 17.7 million fatalities, are CAD and stroke and India accounts for one-fifth of all global mortality, especially among the younger population [6]. India has an age-standardized cardiovascular mortality rate of 272 per 100,000 people, which is much higher than that of other nations [7]. In order to enhance outcomes, patient education is a crucial part of CAD treatment [8,9].

Disease-specific knowledge of CAD patients with CAD is widely studied and reported to be poor [10,11]. It is recommended to develop tailored educational approaches specifically for CAD patients to enhance disease-specific knowledge and thereby improve the outcome of the disease [12,13]. To develop tailored patient education programs, disease-specific knowledge questionnaires are required to assess the knowledge of the patients. Communication between patients and healthcare professionals is facilitated by these educational resources [14].

The CAD Education Questionnaire Short Version (CADE-Q SV) which is a simple tool for assessing CAD knowledge among patients was first created and tested in Brazilian Portuguese before being translated and modified into ten additional languages [15,16]. However, assessment tools for the same in the Marathi language are lacking. In order to give structured and individualized patient education and to test patient understanding, the Marathi version of the questionnaire would be useful. Therefore, the main purpose was to create translated, adapted, and validated Marathi questionnaire of the CADE-Q SV.

## Materials and methods

An observational study was carried out between December 2020 and May 2022 after receiving permission from the Institutional Ethics Committee (IEC) with IEC number EC/NEW/INST/2019/377/16. The standard guidelines provided which required six stages were followed for the translation and modification of the CADE Q-SV [17].

Stage one involved forward translation, in which two bilingual translators, proficient in both Marathi and English that is Translator 1 (T1) & Translator 2 (T2), were identified with the target language as their mother tongue. T1 is an assistant professor in the department of physiotherapy and was familiar with the idea, while T2 did not have any medical background. Both translators provided their translated versions independently, along with a written report of the rationale and choices made during the translation to the study coordinator. Stage two involved the synthesis of the T1 and T2 versions that were performed by the two translators and the study coordinator by working with the initial version of the CADE Q-SV, T1, and T2 versions. The consensus was achieved between the terms and phrases to produce one common version, and T1, and T2 were synthesized.

Stage three consisted of back translation, in which two separate bilingual translators translated the synthesized T1 and T2 versions into English. These translators were completely blinded to the initial version of the CADE Q-SV and did not have any medical background. These versions were named Back T1 (BT1) and Back T2 (BT2). An agreement was then achieved between the terms by comparing the two back-translated versions with the original version. After this stage four included the expert committee, which consisted of the subject expert, study coordinator, research methodologist, two language experts, and the translators who performed forward and back translations of the CADE Q-SV which was given to the committee in both forward and backward translations. The committee drafted the pre-final version by correcting the conceptual terms as per the regional culture and grammatical difficulties.

Stage five involved a pilot study in which the pre-final draft was completed by 30 patients with CAD who visited the outpatient department under the supervision of the study coordinator and their responses were probed. There were 20 items and for every item, the responses were assessed on a Likert scale ranging from 0 to 10. Changes to the pre-final version were made based on the responses recorded by the participants followed by which stage six involved submissions of all reports and final versions to the original author of the study, and approval was obtained.

Test-retest reliability and validity of the Marathi version of the questionnaire were completed by consecutive 200 Marathi-speaking and reading CAD patients visiting the outpatient department of Dr. Bhausaheb Sardesai Talegaon Rural Hospital, Talegaon Dabhade, India twice with an interval of two weeks were recruited using a convenient sampling method. Twelve participants dropped out of the study because they could not report due to transportation and health-related issues. The CADE-Q SV consisted of 20 items; therefore, 200 participants were recruited, considering the thumb rule of 10 participants per item for test-retest validity and reliability.

The CADE-Q SV is a scientifically created and psychometrically verified questionnaire that evaluates the disease-specific knowledge of cardiac patients in the context of secondary prevention factors. There are 20 questions total, and they are grouped into four knowledge domains: medical condition, adverse outcomes, physical fitness, nutrition, and psychosocial risk. Each question has three possible responses: true, false, and I don't know. The highest potential score was 20, four by domain, and one per question. This questionnaire can be used to customize educational sessions for cardiac patients [16,18].

Internal consistency with Cronbach’s alpha (α) and inter-rater reliability with Cohen’s kappa (k) was used to test the psychometric properties. With the aid of the Statistical Product and Service Solutions (SPSS) (IBM SPSS Statistics for Windows, Version 20.0, Armonk, NY) software, data input and analysis were completed. To represent overall knowledge descriptive analysis was done and a total mean score was determined.

## Results

The pre-final questionnaire was given to 30 CAD individuals for testing translation and cultural adaptability. They were asked to complete the questionnaire and probing was done about their understanding. All participants rated a score of 10 as very clear on the Likert scale for all items. Item number 14 to understand the term “Vegetable shortening”, Vanaspati Oil was quoted as an example. This was done in consultation with the expert committee. There were 200 participants in the first session of which 153 were male population (mean 66.31 years) and 47 were women (mean 67.32 years), and 188 participants were in the second session as twelve participants dropped out of the study because they could not report due to transportation and health-related issues (Table 1).

**Table 1:** Number of participants involved in the study. N = number of participants

Total No. of Patients	N = 200
Participants in 1st session	N = 200
Participants in 2nd session	N = 188

Data entry and analysis of test-retest reliability and internal consistency were done using the SPSS software program. The proportion of agreement above and beyond the agreement anticipated by chance was calculated using k. Table 2 shows that κ, a measure of agreement above and beyond the chance agreement, was 1.000 and ranged from -1 to +1. According to Altman's recommendations, which were modified from Landis and Koch's norms [18,19], a κ of 1.000 indicates an extremely high level of agreement. The κ coefficient is statistically substantially different from zero because p < 0.001.

The test-retest reliability of the questionnaire is evaluated on basis of 188 participants' responses during the first and second interview rounds. The results are measured using κ coefficient and overall results meet the minimum recommended standards, as described in Table 2.

**Table 2:** Symmetric measures. a = refusing to hold the null hypothesis, T^b^ = using the null hypothesis and the asymptomatic standard error, n = number of valid cases, k = Cohen's kappa

		Value	Asymptomatic. Standard. Error^a^	Approximate. T^b^	Approximate Significance.
Measure of Agreement (κ)		1.000	0.001	32.605	0.001
Valid Cases (n)		188			

A group of test items' internal consistency or reliability was assessed using α for overall items in which the strength and consistency were evaluated. The Marathi version of the questionnaire demonstrated acceptable test-retest reliability with α value of 0.624 which is at an acceptable level as shown in Table 3.

**Table 3:** Internal consistency statistics.

Cronbach's Alpha	Number of Items
0.624	20

## Discussion

The goal was to translate, modify, and evaluate the Marathi language version of the CADE-Q SV for validity and reliability. The questionnaire was translated into the Marathi language by following standard guidelines. The pre-final version was tested on 30 diagnosed cases of CAD, and final changes were made based on the feedback received from the participants. The final version's validity and reliability were examined. In total, 200 diagnosed cases of CAD were recruited from the outpatient department of Dr. Bhausaheb Sardesai Talegaon Rural Hospital, Talegaon Dabhade, India, and they completed the questionnaire twice at two-week intervals and the participants were able to understand all the components of the questionnaire. Collected data were tested with Cohen's kappa (κ) and Cronbach's alpha (α) to check agreement and reliability, respectively. The Marathi form of the CADE-Q SV demonstrated acceptable test re-test reliability with a Cronbach's alpha value of 0.624. Cohen's kappa value assessing agreement was 1, which is the amount of agreement over and above chance agreement.

The findings of this study mostly agreed with those made available in the initial validation of CADE-Q SV and other languages [15,16,20]. This involved a previous study that involved the original version of the CADE-Q with α value of 0.68 thus demonstrating the accurate validity and reliability of the questionnaire [21]. Similarly, a previous study culturally adopted and validated the Persian version of the CADE-Q and demonstrated that CADE-Q can be accepted in terms of psychometric properties and the questionnaire is easy to understand for doctors as well as for patients [22]. Additionally, a study with α value ranging from 0.70 to 0.81 concluded that the Portuguese version of CADE-Q SV proved to have strong psychometric properties, providing preliminary evidence of its validity and reliability to assess cardiovascular patients' knowledge in Brazil [15]. Furthermore, the questionnaire was also formulated in the Chinese version which showed a validity of 0.94 for the questionnaire and concluded that the Chinese version had strong psychometric properties which made it a useful tool to assess the knowledge of patients on CAD [23].

Knowledge of the disease is a crucial CAD prognostic factor. The lack of disease-specific self-report measures in regional languages is a major barrier to the assessment of disease-specific knowledge of individuals and the implementation of specific goal-based structured patient education programs. The CADE-Q SV Marathi version will be used as an accurate and valid tool to assess an individual’s base knowledge of CAD and implement individualized structured patient education programs.

Because there could be a selection bias, it is important to analyze the findings of the study with caution, as the participants were recruited from only one tertiary health care center who were mainly Marathi-speaking. Furthermore, baseline educational qualifications and socioeconomic criteria were not considered during recruitment, and finally, as the Marathi language has diverse dialects and a vast geographical regional population speaking the language, there can be a few contextual differences while addressing the questionnaire to the entire region, which can be addressed by closely monitoring the individual while completing the questionnaire. Future studies are required to overcome these limitations. However, it is important to test the questionnaire with diverse participants from different demographics, and educational levels to confirm the generalizability of the tool. Also, to the best of our knowledge, there is no standard Marathi CAD knowledge questionnaire previously verified, so we were not able to compare the Marathi version of CADE-Q SV with standard questionnaires to obtain concurrent validity.

## Conclusions

The Marathi version of the CADE-Q SV demonstrated acceptable test-retest reliability with a Cohen's kappa value measuring agreement of 1, which is the proportion of agreement whereas, Cronbach's alpha value was 0.624. Hence, the Marathi version of the questionnaire is cross-culturally adapted and reliable, and, therefore, proves to be an instrumental questionnaire for assessing disease-specific knowledge of CAD among Marathi-speaking individuals.
